# Beta-blocker use and COPD mortality: a systematic review and meta-analysis

**DOI:** 10.1186/1471-2466-12-48

**Published:** 2012-09-04

**Authors:** Mahyar Etminan, Siavash Jafari, Bruce Carleton, John Mark FitzGerald

**Affiliations:** 1 Therapeutic Evaluation Unit, British Columbia Provincial Health Services Authority, Vancouver, Canada; 2 Child & Family Research Institute, University of British Columbia, Vancouver, Canada; 3 Department of Medicine, University of British Columbia, Vancouver, Canada; 4 Division of Respiratory Medicine, University of British Columbia, Vancouver, Canada; 5 School of Population and Public Health, Faculty of Medicine, University of British Columbia, Vancouver, Canada; 6 Division of Translational Therapeutics, Department of Pediatrics, University of British Columbia, Vancouver, Canada; 7 Child & Family Research Institute, A4-198 WS 2, 709-650 West 28th Avenue, Vancouver, BC, V5Z-4H4, Canada

**Keywords:** Beta-blockers, COPD, Mortality

## Abstract

**Background:**

Despite the benefits of beta-blockers in patients with established or sub-clinical coronary artery disease, their use in patients with chronic obstructive pulmonary disease (COPD) has been controversial. Currently, no systematic review has examined the impact of beta-blockers on mortality in COPD.

**Methods:**

We systematically searched electronic bibliographic databases including MEDLINE, EMBASE and Cochrane Library for clinical studies that examine the association between beta-blocker use and all cause mortality in patients with COPD. Risk ratios across studies were pooled using random effects models to estimate a pooled relative risk across studies. Publication bias was assessed using a funnel plot.

**Results:**

Our search identified nine retrospective cohort studies that met the study inclusion criteria. The pooled relative risk of COPD related mortality secondary to beta-blocker use was 0.69 (95% CI: 0.62-0.78; I_2_=82%).

**Conclusion:**

The results of this review are consistent with a protective effect of beta-blockers with respect to all cause mortality. Due to the observational nature of the included studies, the possibility of confounding that may have affected these results cannot be excluded. The hypothesis that beta blocker therapy might be of benefit in COPD needs to be evaluated in randomised controlled trials.

## Background

Beta-blockers are one of the most prescribed classes of cardiovascular medications. In clinical trials they have been shown to lower morbidity and mortality secondary to congestive heart failure [1] (CHF) and coronary artery disease (CAD) [2]. Chronic Obstructive Pulmonary Disease (COPD) is a progressive debilitating lung disease and currently the third leading cause of death in North America [3]. Many patients with COPD also have concomitant CAD. There has been uncertainty with regard to using beta-blockers in COPD patients mainly because of concerns that they might induce bronchospasm and worsen lung function [4], especially in those patients who have a combination of asthma and COPD.

Despite these concerns, there is emerging evidence that beta-blockers may be beneficial in patients with COPD [5,6]. In one systematic review of 19 randomised controlled trials that included patients with both asthma and COPD, beta-blockers lowered FEV_1_ by 7.46% (95% CI, 5.59%-9.32%) [4]. Results from this meta-analysis prompted guidelines to recommend the use of low dose beta-blockers in COPD patients [7].

Recent studies have also shown that beta-blockers may lower mortality in COPD patients [8,9]. This potential benefit may arise from a possible protective effect of beta-blockers in patients with established CAD in whom these drugs may be underused. Beta-blockers may also be beneficial in patients with CAD and overlapping CHF. Finally, similar to statins, beta-blockers, especially cardio-selective beta-blockers such as carvedilol, may exert pleiotropic effects including antioxidant and alpha-adrenorecptor blocking properties [10]. As of to date, no systematic review specifically addressing mortality benefit with beta-blockers in COPD patients has been conducted. We sought to address these questions with respect to a clinically important outcome mainly all cause mortality by undertaking a systematic review and meta-analysis.

## Methods

### Search strategy

We searched the following databases to identify pertinent studies that examined the association between beta-blockers and all cause mortality: MEDLINE (1966 to March 2012), EMBASE (1980 to March 2012), Cochrane Central Register of Controlled Trials (1991 to March 2012), Database of Abstracts of Reviews of Effects (1991 to March 2012), ACP Journal Club (1991 to March 2012), International Pharmaceutical Index (1970 to March 2012), BIOSIS Previews (1969 to March 2012) and Web of Science (1961 to March 2012). The initial search strategy was developed from the following MeSH subject headings COPD, Acebutolol, Atenolol, Betaxolol, Bisoprolol, Celiprolol, Esmolol, Metoprolol, Alprenolol, Bucindolol, Carteolol, Carvedilol, Labetalol, Nadolol, Penbutolol, Pindolol, Propranolol, Sotalol, Timolol, adrenergics, and Beta-blockers in MEDLINE. We reviewed titles for relevance from this search and examined all subject headings and abstracts. The scope notes in MEDLINE and EMBASE were also examined to ensure the correct subject headings were used based on their definitions; other subject headings were included based on previous indexing and the inclusion of keywords based on synonyms used in the scope notes. Proceedings and conference abstracts were searched through the databases PapersFirst (1993) and ProceedingsFirst (1993) up to March 2012. Authors’ names and year of published work from key papers were entered into the cited reference search in the Web of Science. We screened the references of retrieved studies and review articles for any potentially missed articles. In addition, we hand searched the reference lists of retrieved studies as well as journals related to “pharmacology”, “respirology”, pulmonary”, abstracts and books related to respiratory or lung diseases. There was no language restriction in selecting the studies.

### Selection criteria

We considered all experimental or observational studies that assessed the association between beta-blocker use and mortality in COPD patients. Studies were included if they 1) clearly defined COPD as either primary or secondary outcomes; 2) clearly defined beta-blocker use as either a primary exposure in the study or used in a sub-group of patients with COPD; 3) presented relative risks or odds ratios (for all cause mortality) and their corresponding confidence intervals or provided enough data to compute these parameters.

### Data extraction

Included articles were reviewed in full by two reviewers independently (ME and SJ). Study characteristics included in the data extraction form were as follows: authors’ names, publication year, country of study, study design, sample size, study population type, mean or age range, gender of participants, type of risk factors or confounders adjusted for, outcome of the interest (mortality reduction), adjusted odds ratio or relative risks (RRs), and 95% confidence intervals (CIs).

### Statistical analysis

The primary analysis examined the association between beta-blocker (both selective and non-selective) use and mortality in COPD patients. We weighted the study-specific adjusted relative risks (RRs) for cohort studies by the inverse of their variances. Due to the observational nature of the studies, we used the random effects model to estimate the pooled adjusted RR. Statistical heterogeneity between studies was evaluated with Cochran's Q test and the I_2_ statistic. Sensitivity analysis was assessed using the Jackknife procedure by looking at the individual influence of a study and then repeating the analysis by excluding the studies with the largest weights.

## Results

**Figure 1:**
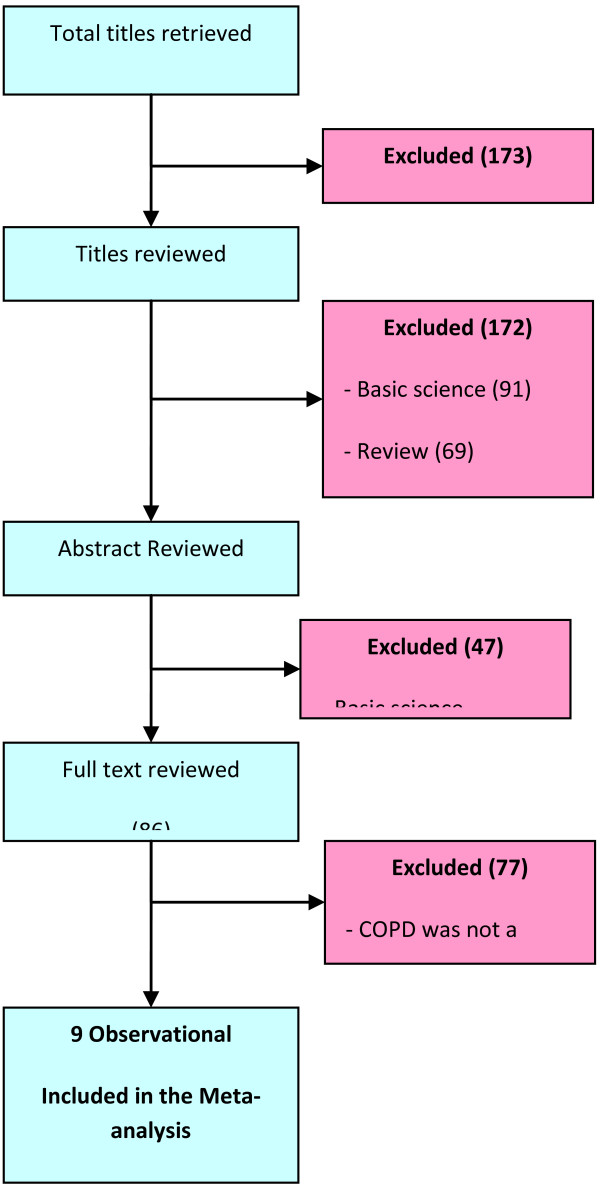
Selection of studies for inclusion in the systematic review and meta-analysis.

**Figure 2:**
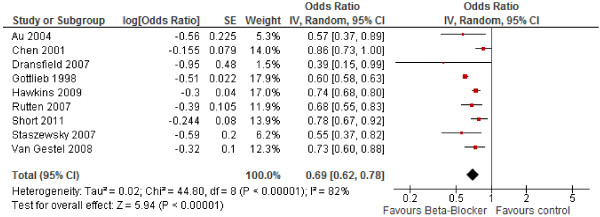
Forest plot of association between beta-blockers and COPD mortality.

**Figure 3:**
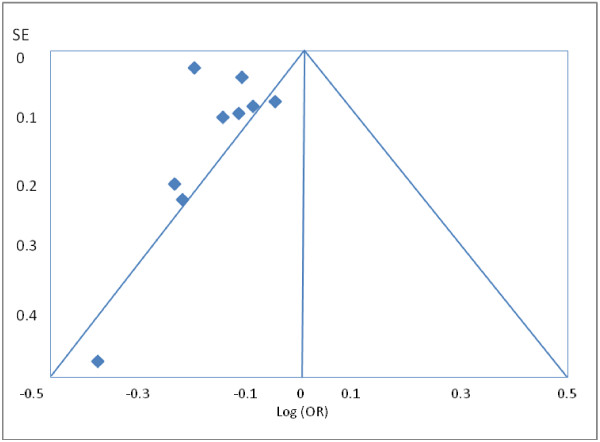
Funnel plot of studies of beta-blocker use and mortality in patients COPD.

Our search resulted in nine retrospective cohort studies [5,6,8,9,11-15] that met our inclusion criteria (Figure 1). Five studies looked at subjects with COPD [11-15] who also had vascular disease or chronic heart failure (Table 1). Three studies looked at concurrent beta-blocker and beta agonist use [8,9,13]. The pooled relative risk of COPD related mortality secondary to beta-blocker use was 0.69 (95% CI: 0.62-0.78; I_2_=82%, p-value=0.00001). Our results indicate a high degree of heterogeneity among the included studies (Figure 2). In a sensitivity analysis, we identified one study [11] as the source of heterogeneity. Exclusion of this study from the analysis removed the study heterogeneity (I_2_=29%, P=0.20) while the pooled RR stayed significantly protective (RR=0.74, 95% CI=0.70-0.79). Examination of the funnel plot revealed presence of publication bias. The funnel plot clearly demonstrates that studies with a null or positive association which should appear symmetrically on the right axis to form the funnel are missing (Figure 3). This means that studies that did not find a protective association for beta-blockers and mortality in COPD patients (negative studies) were less likely to be published.

**Table 1:** Characteristics of included studies in the systematic review

**Study**	**Design**	**Sample size and cohort description**	**Type of BB**	Relative Risk*	**Covariates**
Short (2011) [8]	Retrospective Cohort	5,977 COPD patients in Scotland	Cardio-selective	0.78 (0.67-0.92)	CAD and Respiratory disease, age, sex, diabetes, smoking, FEV1, cardiovascular drugs
Au (2004) [6]	Retrospective Cohort	1966-Veteran Affairs Cohort with COPD	Not specified	0.57 (0.33-0.89)	Comorbidity, age, history of COPD, bronchodilators, smoking, coronary artery disease, diabetes
Dransfield (2008) [5]	Retrospective Cohort	825 subjects admitted to hospital for COPD	Not specified	0.39 (0.14-0.99)	Age, CHD, CHF, Liver disease, COPD exacerbations, malignancy, smoking, FEV
Rutten (2007) [9]	Retrospective Cohort	Electronic records of 2,230 patients with COPD in the Netherlands	Selective and non-selective	0.68 (95% -0.56-0.83), for all BBs: 0.67 (0.55-0.83) for B1 selective BBs. 0.82 (0.61-1.10) for non-selective BBs	Age, sex, diabetes, hypertension, CAD, CVD drugs, pulmonary drugs
Gottlieb (1998) [11]	Retrospective Cohort	41,814 COPD subjects with previous history of myocardial infarction	Not specified	0.60 (0.57-0.63)	Not specified
Staszewsky (2007) [12]	Retrospective Cohort	628 subjects with class II-IV Heart Failure and COPD	Not specified	0.55 (0.37-0.82)	Not specified
Van Gestel (2008) [13]	Retrospective Cohort	1,205 with vascular disease and COPD in Netherlands	Cardio-selective	0.73 (0.60-0.88)	Age, sex, hypertension, hypercholesterolemia, diabetes, renal function, smoking, BMI, CAD, FEV1, cardiovascular drugs
Chen (2001) [14]	Retrospective Cohort	43,974 subjects with previous history of myocardial infarction and COPD or Asthma	Not specified	0.86 (0.73-1.0)	Age, gender, co-morbidities, CAD, cardiovascular drugs, physician speciality
Hawkins (2009) [15]	Retrospective Cohort	1,258 patients with COPD and previous history of MI	Not specified	0.74 (0.68-0.80)	Not specified

* = Relative risk and 95% confidence interval for beta-blocker use and mortality in COPD.

## Discussion

The results of this systematic review are consistent with a mortality benefit with beta-blockers in subjects with COPD. There was a high degree of heterogeneity amongst the studies. The source of heterogeneity was found to be from the study by Gottlieb et al [11]. The study by Gottlieb et al [11] was a retrospective analysis of those taking beta-blockers who had a previous history of myocardial infarction. The authors only reported a rate ratio of mortality amongst users of beta-blockers with a diagnosis of COPD. It is unclear from this study whether this reported rate ratio was adjusted for potential confounders.

Beta-blockers are a popular class of cardiovascular drugs used for multiple cardiovascular conditions including hypertension, congestive heart failure and management of ischemic heart disease. The possibility of bronchospasm with beta-blocker therapy in COPD may have discouraged some clinicians from prescribing these drugs to their COPD patients especially those with concomitant cardiovascular disease. In recent years, there has been emerging literature pointing toward a protective effect from beta-blockers in COPD [5,6,8]. A systematic review by Salpeter and colleagues assessed the effect of beta-blockers in randomised trials of patients with asthma or COPD [4]. The review found that beta-blockers decreased FEV_1_ by 7.46% (95% CI, 5.59%-9.32%) and increased response to beta agonists by 4.63% (95% CI, 2.47%-6.78%) [4]. The limitation of this review was that it included patients with both asthma and COPD***.*** A recent review completed by the same investigators failed to show any benefit or harm with cardioselective beta-blockers on FEV_1_ in COPD patients [16].

The mortality benefits of beta-blockers in COPD are thought to be due to the cardioprotective effect of the drugs. Two other postulated mechanisms may also explain the potential benefits of beta-blockers in COPD. Increased beta-agonist activity has shown to play an important role in the pathology of CHF [17,18]. Thus patients taking beta-blockers with concomitant COPD and CHF may experience a lower degree of beta agonist stimulation.

Finally, it is yet unclear as to whether a protective effect with these drugs is a class-effect or whether this benefit differs with the receptor selectivity (B_1_ selective vs non-selective such as carvedilol). Carvedilol has shown to have pleiotropic properties including antioxidant exerting effects [10] and has shown to lower mortality in patients with CHF. Thus, carvediolol’s mortality reduction in COPD patients may be more profound than that of beta-selective beta-blockers. It is possible that the beneficial effects of beta-blockers go beyond their cardioprotective properties. Heindl et al. examined the sympathetic nerve activity in six COPD patients with no previous history of coronary artery disease and six healthy controls [19]. COPD subjects showed a significantly higher peripheral sympathetic activity than the controls. Rutten et al. assessed the effect of beta-blockers in a sub-group of COPD patients with less severe coronary artery disease [9]. The relative risk of mortality amongst COPD patients who used beta-blockers and only had hypertension was similar to those with more severe form of coronary artery disease (RR=0.67, 95% CI: 0.45-0.99: RR=0.68, 95% CI: 0.56-0.83 respectively).

This review, as with all systematic reviews of observational studies, has limitations. None of the studies were randomised trials and although statistical adjustment was used in all the studies to control for potential confounders, not all confounding variables could have been adjusted in some of the studies. Information on beta-blocker use was also incomplete in most of the studies and did not provide information on the patterns of exposure including, adherence or a dose–response relation for beta-blockers in their relation to COPD mortality. Moreover, many of the studies did not describe in detail how mortality data was obtained. Finally, several types of biases specific to pharmacoepidemiologic studies of respiratory disease may have potentially affected the results of the studies included in this review. We briefly discuss four types of biases including immortal time bias, immeasurable time bias, calendar time bias and confounding by contraindication.

### Selection bias

Selection bias refers to systematic differences between the exposed and unexposed groups in a cohort study. One example of study that may have been affected by selection bias is the study by Au et al [6]. In this study, antihypertensive use was defined as adherence to a medication of 80% or more during the 90 days prior to the event date [6]. Beta-blockers are generally less tolerated than calcium channel blockers mainly due to a less favourable adverse events profile. Thus beta-blocker users may have been ‘healthier’ than calcium channel blocker users and hence may have had a lower overall mortality.

### Immortal time bias

Immortal time bias, first described by Suissa [20], refers to a type of bias that arises mainly from pharmacoepidemiologic studies that use health claims databases. The bias occurs when there is a period of time where drug exposure information prior to hospitalization is missing, as health claim databases usually do not capture in-hospital prescription drug data. Since cases are more likely to experience multiple hospitalizations possibly leading to death, they are less likely to be prescribed a beta-blocker in the period prior to death than control patients [20]. Thus, a lower probability of exposure amongst the cases may lead to a biased protective rate ratio. In this review, the study by Dransfield et al [5] had access to in-hospital medication which makes the possibility of this bias unlikely. However, in the studies that used health claim databases [6,11] the possibility that immortal time bias may have influenced the study results was not discussed and thus cannot be excluded.

### Calendar time bias

Calendar time bias refers to a bias that may be caused by time-trends that may lead to differential prescribing of one drug over another. For example, the Study by Au et al [6] compared the risk of mortality amongst COPD patients who took beta-blockers to those who took other antihypertensives including calcium channel blockers. Antihypertensive drug therapies especially calcium channel blockers’ prescribing trends may vary in time and may be affected by publication of land-mark trials, hypertension guidelines or changes in policy. Thus changes in antihypertensive prescribing over time may lead to differential prescribing of antihypertensives which may also affect COPD patients.

### Confounding by contraindication

Confounding by indication is possibly the most important type of confounding that threatens the validity of pharmacoepidemiologic studies. This type of confounding refers to a situation where the observed association with a drug is in fact due to the condition for which the drug is used. Confounding by contraindication is inherently the opposite of this situation where the drug is knowingly withheld by a clinician due to concerns that the drug may worsen a patient’s condition. Beta-blocker use in COPD is a classic example where confounding by contra-indication may occur as historically clinicians have been hesitant in prescribing beta-blockers to COPD patients. This type of confounding may explain the protective effect observed with beta-blockers in the COPD population mainly due to a small number of patients with COPD who received beta-blockers compared to other types of antihypertensives. Another possibility is that clinicians may prescribe beta-blockers to those with a less severe form of COPD who may have a lower risk of mortality.

## Conclusion

The mortality benefit from this review is similar in magnitude to that of statins which have been previously studied in this setting [21,22] and are currently being tested in randomised controlled trials [23]. Although the results of this review are consistent with a protective effect of beta-blockers and COPD mortality, several types of biases may have affected these results. We believe that our review generates a strong hypothesis that beta-blockers may have mortality benefit in COPD patients. However, this potential benefit must be critically examined in a large RCT. Patients with underlying COPD who are already receiving beta-blockers for other medical conditions may benefit from beta-blocker therapy. However, initiation of beta-blocker therapy in COPD patients to achieve mortality benefit must be reserved until results from RCTs specifically addressing this question are available.

## Competing interest

The authors declare that they have no competing interests.

## Authors’ contributions

ME had full access to all of the data in the study and takes responsibility for the integrity and accuracy of the data. Study concept and design: ME, SJ, JMF. Acquisition of data: ME, SJ. Analysis and interpretation of data: ME, SJ. Drafting of the manuscript: ME, SJ, JMF. Critical revision of the manuscript: ME, SJ, BC, JMF. Statistical analysis: ME, SJ. Administrative, technical, or material support: ME. Funding/Support: This was an unfunded project. All authors read and approved the final manuscript.

## Pre-publication history

The pre-publication history for this paper can be accessed here:

http://www.biomedcentral.com/1471-2466/12/48/prepub
